# Does the BioBLU 0.3f single-use scale to the BioFlo® 320 reuseable bioreactor on a matched volumetric oxygen mass transfer coefficient?

**DOI:** 10.1007/s11274-020-02968-2

**Published:** 2021-01-04

**Authors:** Williams Olughu, Duncan Galbraith, Cillian Paget, Steve Ruscoe, Josh Smith, Alison Mason

**Affiliations:** Ipsen Biopharm Ltd, Unit 9 Ash Road North, Wrexham Industrial Estate, Wrexham, LL13 9UF UK

**Keywords:** Scale up of fermentation, Scale down of bioprocesses, Bioprocess development, Fermentation, Bioreactor characterization

## Abstract

The volumetric oxygen mass transfer coefficient ($k_{l} a$) is an essential parameter in aerobic high-cell density fermentation where the availability of oxygen to growing microorganisms is a limiting factor. Bioprocess teams looking to scale-up/down between the Eppendorf BioBLU 0.3f single-use vessel and the BioFlo® 320 reusable vessel bioreactors may find it challenging using a matched $k_{l} a$. The maximum $k_{l} a$ of the BioFlo® 320 reusable bioreactor was 109 h^−1^, which was approximately twice that of the BioBLU 0.3f single-use vessel. The results here show no overlap in $k_{l} a$ values when both bioreactors were compared and thus conclude that scalability based on $k_{l} a$ is not viable. The maximum $k_{l} a$ of the Eppendorf BioBLU 0.3f single-use reported here was 47 h^−1^ compared to that of the manufacturer’s value of 2500 h^−1^, indicating a 53-fold difference. This discrepancy was attributed to the incompatible sulfite addition method used by the manufacturer for estimation.

**Electronic supplementary material:**

The online version of this article (10.1007/s11274-020-02968-2) contains supplementary material, which is available to authorized users.

## Introduction

The use of scale-down models (SDM) in bioprocess development is an essential tool for troubleshooting problems of the large-scale (Enfors et al. 2001). For example, in fermentation, a validated high throughput scale-down bioreactor confers the advantage of rapid testing of hypothesis and cost-savings during development to mention but a few (Olughu et al. 2020). Thus, process development teams typically spend considerable time and effort to develop reliable SDM. However, there should be an implicit understanding that all models are fundamentally wrong and the most useful are approximately correct within limited boundaries. Consequently, a reasonable aim of designing an SDM is to create a comparable environment across scale. Therefore, it should be understood that a valid SDM is at best, an approximate of the mimicked large-scale environment.

Likewise scaling up is a useful way of improving commercial viability, due to the reduction of manufacture cost on upscaling (Reisman 1993). Hence, ensuring that the early-stage process development environment at the small-scale is closely replicated at the large-scale industrial phase is crucial for economic success.

The scope of this work is limited to fermentation, which is the primary upstream unit of operation in most bioprocesses. On scale-up/down of fermentation, the principal parameters typically considered are volumetric power input, mixing time, volumetric oxygen mass transfer coefficient ($k_{l} a$) and impeller tip speed (Garcia et al. 2010; Olughu et al. 2019). Of these, the most important or widely used are the volumetric power input and $k_{l} a$, but in fermentations where oxygen limitation is of major concern (especially high-cell density aerobic bioprocesses), scaling-up/down based on a constant $k_{l} a$ becomes the most relevant factor (Garcia et al. 2010).

The $k_{l} a$ combines the resistance coefficient of oxygen transfer from air to liquid medium ($k_{l}$) and the interfacial area ($a$), which is a function of the gas hold-up time and bubble diameter (Van’t Riet 1979). The $k_{l} a$ relationship to the oxygen transfer rate (OTR) and the oxygen uptake rate (OUR) of a growing cell is described by the Eqs. (1–3), which forms one of the foundations of bioreactor design.

1 $$\begin{array}{*{20}c} {\frac{{dC_{L} }}{dt} = OTR - OUR} \\ \end{array}$$

2 $$\begin{array}{*{20}c} {OTR = k_{L} a\left( {C^{*} - C_{L} } \right).} \\ \end{array}$$

Substituting Eq. (2) into Eq. (1) results in

3 $$\begin{array}{*{20}c} {\frac{{dC_{L} }}{dt} = k_{L} a\left( {C^{*} - C_{L} } \right) - OUR} \\ \end{array}$$

C* is the concentration of oxygen at the gas–liquid interface and $C_{L}$ the concentration of oxygen in the liquid phase. From Eq. (3) it can be inferred that the $k_{l} a$ is a measure of the effectiveness of a bioreactor at making oxygen available to the growing cells. Thus, scaling up/down based on $k_{l} a$ intends to replicate similar dissolved oxygen concentrations to the cells across varying scales.

However, even when development scientists decide to scale up/down based on matched $k_{l} a$, there is still a choice to be made about the most appropriate experimental method. The experimental approach adopted to measure the $k_{l} a$ needs to be suitable for the process and equipment capability if significant measurement errors are to be avoided. Some of these experimental methods used in determining the $k_{l} a$ in bioreactors are dynamic gassing-out with and without cells, chemical (addition of sulphite or hydrazine) and gas-phase analysis.

For example, the gas-phase analysis method used to measure the OTR directly can be argued to be an easy setup. The inlet and outlet gas analyser measure the air composition going through the system, and the net difference in oxygen concentration is computed as the OTR. Although simple, its utility is dependent on the accuracy of the mass flow meter, gas analyser and a requirement for large gas flows (Wang et al. 1979). Hence, using such a method would not be suitable for $k_{l} a$ measurements in small-scale bioreactors. In contrast, the dynamic gassing-out method without cells was used here, due to the simplicity and the lack of interference from metabolic activity, which also reduced experimental complexity.

## Materials and methods

The BioBLU® 0.3f single-use and the BioFlo® 320 reusable bioreactors (Eppendorf AG, Germany) were used in this study. M9 medium was used, and the working volumes were 0.18 L and 4 L in the BioBLU® 0.3f and BioFlo® 320 bioreactors, respectively. Table 1 shows the physical design and dimensions of both vessels.

**Table 1:** Physical design of the BioBLU® 0.3f single-use and the BioFlo® 320 reusable bioreactors

	BioBLU® 0.3f single-use	BioFlo® 320 reusable
Vessel height (mm)	150	339
Vessel diameter (mm)	70	185
Impeller type	Rushton	Rushton
Number of impellers	2	2
No. of baffles	None	4
Impeller diameter (mm)	30	80
Impeller width (mm)	7	27.7
Impeller height (mm)	8	6.9
Distance between impellers (mm)	25	65

**Fig. 1:**
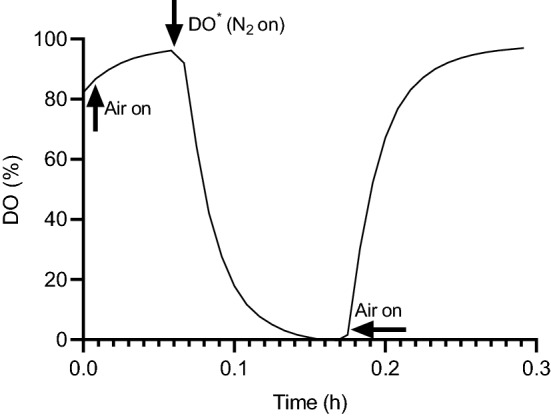
A typical profile of the dissolved oxygen concentration vs. time using the dynamic gassing-out method in the experimental determination of ${k_{l}} a$

As mentioned above, the dynamic gassing-out method without addition of cells was adopted for estimating and comparing the $k_{l} a$ values of both systems. This method can be broadly divided into three stages (Fig. 1). (1) Aeration of the medium until the dissolved oxygen (DO) reaches saturation O*, (2) Stripping off of DO with nitrogen and (3) Re-aeration.

Because no cells were introduced in this method, Eq. (4) satisfies the mass balance of oxygen within the bioreactor on re-aeration of the medium at the final stage.

4 $$\frac{{d\left( {DO} \right)}}{dt} = k_{L} a\left( {DO^{*} - DO} \right)$$

where DO^*^ represents the arbitrary set 100% value dependent on the starting conditions and DO is the value of the dissolved oxygen after the start at any time t. Integrating Eq. (4) from t_0_ to t and DO_0_ to DO results in Eq. (5)

5 $$\ln \left( {DO^{*} - DO} \right) = - k_{l} a\left( {t - t_{0} } \right) + ln\left( {DO^{*} - DO_{0} } \right)$$

Thus, Eq. (5) is a straight line equation and the plot of $\ln \left( {DO^{*} - DO} \right)$ vs $\left( {t - t_{0} } \right)$ results in a slope, which equates $k_{l} a$.

The experimental conditions from which the $k_{l} a$ of BioBLU® 0.3f single-use and the BioFlo® 320 reusable bioreactors were evaluated are given in Table 2. The temperature within the range of 16 °C to 37 °C did not have any significant impact on the estimated $k_{l} a$ values (results shown in the Supplementary section); hence all further experiments were carried out at 26 ± 2 °C. All experimental conditions were replicated twice, and the reagents used were all of an analytical grade.

**Table 2:** Experimental conditions for estimating $k_{l} a$ for the BioBLU® 0.3f single-use and the BioFlo® 320 reusable bioreactors, * vessel maximum agitation rate

BioBLU® 0.3f single-use			BioFlo® 320 reusable		
Run	Aeration (vvm)	Agitation (rpm)	Run	Aeration (vvm)	Agitation (rpm)
1	0.5	350	1	0.5	200
2	1	350	2	1	200
3	2	350	3	2	200
4	0.5	900	4	0.5	400
5	1	900	5	1	400
6	2	900	6	2	400
7	0.5	1400	7	0.5	600
8	1	1400	8	1	600
9	2	1400	9	2	600
10	0.5	2000*	10	0.5	700
11	1	2000*	11	1	700
12	2	2000*	12	2	700
			13	0.5	900
			14	1	900
			15	2	900
			16	0.5	1000
			17	1	1000
			18	2	1000
			19	0.5	1100
			20	1	1100
			21	2	1100
			22	0.5	1200*
			23	1	1200*
			24	2	1200*

## Results

**Fig. 2:**
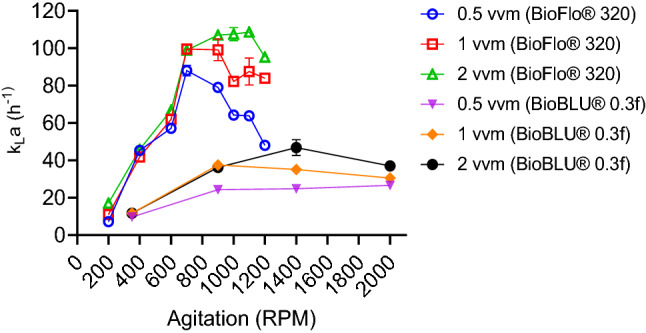
Estimates of $k_{l} a$ values for the BioBLU® 0.3f single-use and the BioFlo® 320 reusable bioreactors. The error bars indicate the standard deviation of two replicates

Figure 2 compares the $k_{l} a$ of the BioBLU® 0.3f single-use and the BioFlo® 320 reusable bioreactors as a function of agitation and aeration rates. Here the average maximum $k_{l} a$ value reached for the BioFlo® 320 was 109 h^−1^ at 2 vvm and 1100 rpm, while that of the BioBLU® 0.3f was 47 h^−1^ at 2 vvm and 1400 rpm. These results infer that within the range studied a higher airflow rate generally led to a higher $k_{l} a$ value. The difference in the maximum $k_{l} a$ valve observed in the BioBLU® 0.3f at 2 vvm compared to 0.5 vvm was approximately twofold; in the BioFlo® 320, this difference was 1.2-fold. The lowest $k_{l} a$ values were seen at the lowest agitation and airflow rates in both systems. The $k_{l} a$ profiles in the BioBLU® 0.3f generally showed an increase with increasing agitation and airflow until after 900 rpm. In comparison, for the BioFlo® 320, this decrease happened after 700 rpm at 0.5 vvm and 900 rpm at 1 and 2 vvm.

## Discussion

Although the $k_{l} a$ values in both systems were quantitatively different, the $k_{l} a$ trends showed some similarities. They illustrate an initial increase in $k_{l} a$ as agitation and airflow rates were increased up to a point before plateauing off (Fig. 2). The results observed in both systems broadly track results from other investigators (Hudcova et al. 1989; Paglianti et al. 2000), the initial bulk flow starts as being flooded, followed by the transition to a loaded regime as agitation and airflow increase. At the peak $k_{l} a$ values seen in Fig. 2, the bulk flow can be said to be dispersed, but towards the end, it transits back to a loaded-flooded bulk flow, hence the decrease seen in $k_{l} a$. An impeller is said to be flooded when the gas phase flows up towards the center of the vessel, while the liquid phase flows up towards the walls (Hudcova et al. 1989). However, as the impeller becomes loaded, its angular velocity increases and the gas phase flows over its cross-sectional area, so the further increase in the impeller angular velocity results in a fully dispersed bulk flow (Hudcova et al. 1989; Paglianti et al. 2000; Bombač and Žun 2006).

The steeper $k_{l} a$ decrease observed in the BioFlo® 320 reactor could be attributed to the transition from a loaded to a flooded regime, due to a significant formation of ragged cavities (Paglianti et al. 2000). However, for the BioBLU® 0.3f, the formation of either vortex/clinging cavities or “3–3” structure remained predominant at high agitation rates; hence the loaded bulk flow persistence. The disparity in bulk flow patterns between the two systems may also be linked to significant differences in the gassed and un-gassed power input values at high agitation rates (Hudcova et al. 1989).

Figure 2 also suggests that scaling up/down based on matched $k_{l} a$ values between both systems is not feasible. The results here show no $k_{l} a$ overlap when both vessels were compared. In contrast, the manufacturer of the BioBLU® 0.3f reported a maximum $k_{l} a$ value of 2500 h^−1^ (Huether-Franken and Kleebank 2014); their results indicate a 53-fold increase compared to that estimated here. This discrepancy in values is difficult to harmonise, but it is most likely due to the use of the sulphite method for evaluating $k_{l} a$, which is known not to be suitable for microbial processes (Garcia and Gomez 2009). The sulphite method changes the diffusion and bubble coalescence characteristics, reduces the complex gas–liquid boundary layer and changes the driving force significantly (Garcia and Gomez 2009). Thus, the interactions of these factors most likely resulted in the grossly overestimated $k_{l} a$ value reported in Huether-Franken and Kleebank (2014).

## Conclusion

The $k_{l} a$ values of the BioBLU® 0.3f and BioFlo® 320 bioreactors have been shown to be different, with no overlap. Hence, scalability on a matched $k_{l} a$ is not viable; other scale-up/down criteria have to be explored if these systems are to be used concurrently in bioprocess development. It is important to state that this discrepancy in $k_{l} a$ reporting may not be unique to the Eppendorf bioreactors studied here. Hence process development scientist should independently confirm the $k_{l} a$ values of their vessels irrespective of the bioreactor manufacturer.

It is also crucial that regardless of the scale-up/down criteria adopted, more fermentation outputs (titre, metabolites, biomass, viability, impurities) should be quantified to confirm process similarity across scales, and thus pivoting away from the single output approach commonly used to verify scalability. The idea of considering only biomass concentration as a measure of success during scale-up/down increases the chance of failure, as the other outputs mentioned above are also crucial for evaluating the physiological state of cells. Disregarding the inclusion of more fermentation outputs encourage the creation of a different environment when a process is scaled up or down, which may consequently lead to a significant difference in product quality, impurities and productivity as the scale is varied.

## Electronic supplementary material

Below is the link to the electronic supplementary material.

Electronic supplementary material 1 (DOCX 30 kb)

## Abbreviations

*C**: Concentration of oxygen at the gas–liquid interface
$C_{L}$: Concentration of oxygen in the liquid phase
$$k_{l}$$: Resistance coefficient
$k_{l} a$: Volumetric oxygen mass transfer coefficient
$$a$$: Interfacial area
SDM: Scale-down models
OTR: Oxygen transfer rate
DO: Dissolved oxygen
DO*: Dissolved oxygen at saturation
t: Time
t_0_: Time at start
OUR: Oxygen uptake rate
