# Supplementary material for: Does the BioBLU 0.3f single-use scale to the BioFlo® 320 reuseable bioreactor on a matched volumetric oxygen mass transfer coefficient?
Source: World J Microbiol Biotechnol. 2021 Jan 4;37(1):11. doi: 10.1007/s11274-020-02968-2 (PMC7779418; doi:10.1007/s11274-020-02968-2)
Supplement: Supplementary file 1 — Electronic supplementary material 1 (DOCX 30 kb) [file 11274_2020_2968_MOESM1_ESM.docx]

Table 3, BioBLU 0.3f single-use vessel experimental overview with temperature taken into consideration for evaluating $k_{l}a$

| Run | Temp (C) | Aeration (vvm) | Agitation (rpm) | Average $k_{l}a$ (h^-1^) |
| --- | --- | --- | --- | --- |
| 1 | 16 | 0.5 | 350 | 8.0 |
| 2 | 26 | 0.5 | 350 | 10.0 |
| 3 | 37 | 0.5 | 350 | 10.1 |
| 4 | 16 | 1 | 350 | 10.5 |
| 5 | 26 | 1 | 350 | 11.7 |
| 6 | 37 | 1 | 350 | 14.2 |
| 7 | 16 | 2 | 350 | 9.3 |
| 8 | 26 | 2 | 350 | 11.7 |
| 9 | 37 | 2 | 350 | 15.3 |
| 10 | 16 | 0.5 | 900 | 31.1 |
| 11 | 26 | 0.5 | 900 | 24.4 |
| 12 | 37 | 0.5 | 900 | 24.3 |
| 13 | 16 | 1 | 900 | 48.0 |
| 14 | 26 | 1 | 900 | 36.8 |
| 15 | 37 | 1 | 900 | 34.6 |
| 16 | 16 | 2 | 900 | 37.6 |
| 17 | 26 | 2 | 900 | 36.2 |
| 18 | 37 | 2 | 900 | 40.9 |
| 19 | 16 | 0.5 | 1400 | 26.7 |
| 20 | 26 | 0.5 | 1400 | 24.9 |
| 21 | 37 | 0.5 | 1400 | 30.0 |
| 22 | 16 | 1 | 1400 | 39.5 |
| 23 | 26 | 1 | 1400 | 35.1 |
| 24 | 37 | 1 | 1400 | 39.7 |
| 25 | 16 | 2 | 1400 | 59.6 |
| 26 | 26 | 2 | 1400 | 46.8 |
| 27 | 37 | 2 | 1400 | 37.5 |
| 28 | 16 | 0.5 | 2000 | 39.2 |
| 29 | 26 | 0.5 | 2000 | 26.7 |
| 30 | 37 | 0.5 | 2000 | 28.6 |
| 31 | 16 | 1 | 2000 | 32.2 |
| 32 | 26 | 1 | 2000 | 30.6 |
| 33 | 37 | 1 | 2000 | 36.6 |
| 34 | 16 | 2 | 2000 | 42.9 |
| 35 | 26 | 2 | 2000 | 37.1 |
| 36 | 37 | 2 | 2000 | 37.3 |

A multi-linear regression analysis with $k_{l}a$ as the dependent variable, while temperature, agitation and aeration rate were the independent variables can be seen in Table 4.

| Parameter estimates | Variable | Estimate | Standard error | 95% confidence interval |
| --- | --- | --- | --- | --- |
| β0 | Intercept | 10.23 | 6.511 | -3.032 to 23.49 |
| β1 | Temperature (C) | -0.1320 | 0.1811 | -0.5010 to 0.2370 |
| β2 | Aeration (vvm) | 6.525 | 2.491 | 1.450 to 11.60 |
| β3 | Agitation (rpm) | 0.01310 | 0.002548 | 0.007912 to 0.01829 |
|  |  |  |  |  |
| Sig. diff. than zero? | Variable | \|t\| | P value | P value summary |
| β0 | Intercept | 1.571 | 0.1260 | not significant |
| β1 | Temperature (C) | 0.7288 | 0.4715 | not significant |
| β2 | Aeration (vvm) | 2.619 | 0.0134 | significant |
| β3 | Agitation (rpm) | 5.142 | <0.0001 | significant |

Table 4, multi-linear regression analyses with $k_{l}a$ as the dependent variable

Table 4 indicates that within the range investigated; the temperature had no significant effect on the $k_{l}a$ value.
